# TNIK-driven regulation of ERK5 transcriptional activity in endothelial cells

**DOI:** 10.3389/fcvm.2025.1526676

**Published:** 2025-07-02

**Authors:** Venkata Subrahmanya Kumar Samanthapudi, Sivareddy Kotla, Nhat-Tu Le

**Affiliations:** ^1^ Department of Cardiology, The University of Texas MD Anderson Cancer Center, Houston, TX, United States; ^2^ Academic Institute, Department of Cardiovascular Sciences, Center for Cardiovascular Sciences, Weill Cornell Medical College, Houston Methodist Research Institute, Houston, TX, United States

**Keywords:** ERK5, TNIK, transcriptional activity, inflammation, endothelial cells

## Abstract

Extracellular signal-regulated kinase 5 (ERK5) is essential for cardiovascular development and endothelial cell (EC) function. Activation of ERK5 through MEK5-mediated phosphorylation at threonine 218 and tyrosine 220 (T218/Y220) drives the transcriptional activation of myocyte enhancer factor-2 (MEF2), promoting the expression of *KLF2* and *KLF4—*key transcription factors that maintain vascular homeostasis. We previously demonstrated that ponatinib suppresses ERK5 transcriptional activity without affecting laminar-flow (l-flow)-induced T218/Y220 phosphorylation, suggesting a non-canonical regulatory mechanism. Since ponatinib inhibits Traf2- and Nck-interacting kinase (TNIK), we hypothesized that TNIK modulates ERK5 transcriptional activity. Using a mammalian one-hybrid assay and quantitative RT-PCR (qRT-PCR), we show that TNIK knockdown reduces ERK5 transcriptional activity and downregulates *KLF2*, *KLF4*, and *eNOS* expression, whereas TNIK overexpression enhances ERK5 transcriptional activity. Constitutively active MEK5 (CA-MEK5*α*) rescues ERK5 transcriptional activity in TNIK-depleted cells, but TNIK overexpression fails to overcome inhibition by dominant-negative MEK5 (DN-MEK5), indicating a MEK5-dependent mechanism. Moreover, phosphorylation-deficient TNIK mutants (S764A and S769A) retain the ability to enhance ERK5 transcriptional activity, suggesting a kinase-independent regulatory role. TNIK knockdown also increases NF*κ*B activity and EC apoptosis, linking TNIK to the regulation of inflammatory and survival pathways. These findings identify TNIK as a novel modulator of ERK5 signaling through both MEK5-dependent and independent mechanisms, highlighting its potential as a therapeutic target for vascular inflammation and endothelial dysfunction.

## 1 Introduction

Extracellular signal-regulated kinase 5 (ERK5), composed of an N-terminal kinase domain and a C-terminal transcriptional activation domain (1, 2), is critical for cardiovascular development and endothelial cell (EC) homeostasis by regulating survival, proliferation, and anti-inflammatory responses (3–12). In ECs, ERK5 promotes vascular protection by upregulating endothelial nitric oxide synthase (eNOS), suppressing adhesion molecule expression, and reducing leukocyte-endothelial interactions (2, 13). Canonical activation of ERK5 involves dual phosphorylation at threonine 218 and tyrosine 220 (T218/Y220) within its activation loop by mitogen-activated protein kinase kinase 5 (MEK5). This phosphorylation induces a conformation change that facilitates further phosphorylation within the C-terminal transcriptional activation domain and nuclear translocation, enabling ERK5 to activate myocyte enhancer factor-2 (MEF2). MEF2, in turn, promotes the expression of *KLF2* and *KLF4*, transcription factors essential for maintaining vascular homeostasis (1, 4–8, 14). The MEK5-ERK5-MEF2 axis mediates the anti-inflammatory and vasoprotective effects of laminar flow (l-flow), thereby preserving vascular integrity (12, 15, 16).

Beyond its canonical MEK5-dependent activation (1, 4–8, 14), ERK5 can regulate gene transcription independently, suggesting the existence of additional regulatory mechanisms (2). Indeed, ERK5 activity can be modulated by non-canonical pathways. Our previous studies demonstrated that EC-specific ERK5 deficiency reduces eNOS expression, increases pro-inflammatory molecule expression [e.g., vascular cell adhesion molecule-1 (VCAM-1) and E-selectin], and exacerbates EC dysfunction (3). Furthermore, pro-inflammatory conditions inhibit ERK5 transcriptional activity through p90 ribosomal S6 kinase (p90RSK)-mediated phosphorylation at serine 496 (S496) (3), and small ubiquitin-like modifier (SUMO) conjugation at lysines K6 and 22 (K6/K22) further suppresses ERK5 transcriptional function (11). Additionally, regulation of ERK5 independent of C-terminal phosphorylation or nuclear localization has been reported (8). Collectively, these findings highlight that both canonical and non-canonical mechanisms regulate ERK5 activity, underscoring its multifaceted role in maintaining vascular homeostasis. However, the complete regulatory network controlling ERK5 transcriptional activation remains incompletely understood, warranting further investigations.

Traf2- and Nck-interacting kinase (TNIK), a serine/threonine kinase within the Ste20 kinase family (17–19), comprises an N-terminal kinase domain, an intermediate domain, and a C-terminal regulatory domain that mediates interactions with proteins such as Rap2, Traf2, and Nck (20). TNIK is involved in actin cytoskeleton remodeling through phosphorylation of gelsolin (17) and regulates β-catenin/T-cell factor-4 (TCF-4) transcriptional complexes within the Wnt signaling pathway (18, 21–23). It has been implicated in Wnt-driven proliferation in colorectal cancer and leukemia (18, 21–23) and contributes to tumorigenesis in lung squamous cell carcinoma by phosphorylating TCF4/TCF7L2 and neurofibromin 2 (NF2) at serine 13 (S13) (18, 21, 24–28). Beyond its oncogenic roles, TNIK regulates neuronal synaptic structure and function (20, 29–38). Genome-wide association studies and functional analyses have linked TNIK to psychiatric disorders, with TNIK knockout mice exhibiting hyper-locomotor behavior reversible by glycogen synthase kinase 3β (GSK3β) inhibitors (29, 32, 39–41). TNIK also activates c-Jun N-terminal kinase (JNK) within the mitogen-activated protein kinase (MAPK) family, although its regulatory effects on other MAPK members, such as ERK and p38, remains less well characterized (17, 20). Despite these diverse roles in cancer and neural systems, the function of TNIK in vascular biology remains incompletely understood.

Ponatinib, a third-generation tyrosine kinase inhibitor used to treat chronic myeloid leukemia harboring the Bcr-Abl T315I mutation, is highly effective but induces severe vascular adverse events that limit its clinical utility. These adverse effects include EC dysfunction, apoptosis, and impaired angiogenesis, partly through inhibition of vascular endothelial growth factor receptor 2 (VEGFR2), contributing to systemic and pulmonary hypertension (42, 43). Our previously studies demonstrated that ponatinib increases NFkB p65 phosphorylation and activity, elevates inflammatory gene expression, disrupts barrier integrity, and promotes apoptosis in ECs. Importantly, ponatinib suppresses ERK5 transcriptional activity and the expression of its target genes (*KLF2, KLF4, eNOS*) even under conditions of constitutively active MEK5α (CA-MEK5α) overepxression (44). Notably, ponatinib also inhibits TNIK (45). These findings suggest that TNIK may play a regulatory role in modulating ERK5 transcriptional activity.

Using RNA-sequencing (RNA-seq) and pathway enrichment analyses, we previously demonstrated that TNIK regulates interferon signaling and cytokine responses in ECs (46). However, its role in ERK5 transcriptional regulation remains unexplored. Here, employing a mammalian one-hybrid assay to evaluate ERK5 transcriptional activation (3, 11, 47) and quantitative RT-PCR (qRT-PCR) (48) to quantify gene expression, we sought to elucidate the mechanisms by which TNIK modulates ERK5 transcriptional activity in ECs.

## 2 Methods

### 2.1 EC culture

Human aortic endothelial cells (HAECs) were generously provided by Dr. Aldons J. Lusis (49) (University of California, Los Angeles, David Geffen School of Medicine). These cells were isolated from aortic explants obtained from multiple heart transplant donors and thoroughly characterized, as detailed in Romanoski et al. *(*49). Human umbilical vein endothelial cells (HUVECs) were isolated from umbilical cord veins by collagenase digestion and cultured on gelatin-coated dishes (0.2%; MP Biomedicals, #901771) in endothelial cell medium (ECM; Science Cell, #1001) according to established protocols (44, 46, 50).

All experimental protocols were approved by the Houston Methodist Research Institute Institutional Review Board (IRB Pro00020559). Informed consent was not required for the use of cells from de-identified donor tissues. While most experiments were conducted using HAECs, preliminary studies indicated no significant differences in experimental trends between HAECs and HUVECs. Therefore, HUVECs were utilized in certain experiments when HAECs were unavailable. All ECs used in this study were between passages 4 and 6.

### 2.2 L-flow generation using a cone-and-plate apparatus

L-flow was generated using a cone-and-plate apparatus, a system routinely employed in our laboratory to study EC responses to physiological shear stress. The device consists of a smooth, polished cone positioned above a culture plate seeded with ECs. Rotation of the cone at a constant speed, driven by a magnetic plate system, produced a uniform laminar shear stress across the EC monolayer.

For l-flow experiments, the rotation speed and cone angle were meticulously calibrated according to established laboratory protocols to generate a shear stress of approximately 12 dyne/cm^2^, mimicking the physiological hemodynamic environments experienced by ECs in arterial regions characterized by unidirectional flow. Strict adherence to calibration procedures ensured consistent and reproducible shear stress across the culture surface.

Under l-flow conditions, ECs exhibited characteristic morphological adaptations, including elongation, spindle-shaped morphology, and alignment in the direction of flow. This morphological response serves as a hallmark indicator of effective laminar shear stress and faithfully recapitulates the phenotype of ECs in healthy arterial regions *in vivo*.

### 2.3 Generation of TNIK wild-type (WT) and phosphorylation site mutants (S764a and S769a)

The TNIK WT construct was generated by cloning the TNIK open reading frame (ORF) (NM_015028, SKU RC224180; Origene) into the pCMV-Tag2B expression vector using KpnI and XhoI restriction sites. The pCMV-Tag2b vector contains a cytomegalovirus (CMV) promoter for robust mammalian expression, a FLAG tag for protein detection, and SV40 and HSV-TK polyadenylation signals to enhance transcript stability. Following ligation, plasmids were transformed into *Escherichia coli* DH5*α* cells and selected on kanamycin-containing plates. Proper insert orientation and sequence integrity were verified by sequencing (Snap Gene software).

Site-directed mutagenesis was performed to generate TNIK phosphorylation site mutants (S764A and S769A). Primers were designed to substitute alanine for serine at positions 764 or 769, respectively, within the TNIK coding sequence. Mutagenesis was conducted using the pCMV-Tag2B-TNIK WT plasmid as a template, and mutant constructs were transformed into *E. coli* XL10-Gold cells, followed by kanamycin selection. Successful incorporation of the intended mutations was confirmed by sequencing.

The resulting constructs, pCMV-Tag2B-TNIK S764A and pCMV-Tag2B-TNIK S769A, retained all essential features of the parental vector, including the CMV promoter, FLAG tag, and kanamycin resistance cassette, ensuring reliable and consistent expression of WT and mutant TNIK proteins in subsequent mammalian cell experiments.

### 2.4 siRNA-mediated TNIK knockdown and TNIK overexpression

TNIK-specific small interfering RNA (siRNA) (siTNIK), targeting nucleotides 843–857 of the human TNIK mRNA sequence (5′-CuAAGGAuGuGGuGCuCCA-3′), and a non-targeting control siRNA (siCont: 5′-AACACAGuGGAGCGAAuuCCu-3′) (46) were purchased from Sigma-Aldrich. ECs were transfected with either siTNIK or siCont (50 nM each) using Lipofectamine reagent (Invitrogen), following the manufacturer's protocol optimized for EC transfection.

For TNIK overexpression, ECs were transfected with 2 µg of TNIK expression plasmid per 10⁷ cells in Opti-MEM reduced serum medium (Thermo Fisher Scientific, #31985070) using Plus reagent (Life Technologies, #11514015) in combination with Lipofectamine (Life Technologies, #18324020) (51). To ensure consistency across conditions, total siRNA or plasmid amounts were normalized by supplementing with siCont or empty vector, respectively, as needed.

Cells were harvested for downstream analyses at 48 h post-siRNA transfection or 24 h post-plasmid transfection, depending on the specific experimental design (3, 46, 51). These time points were selected based on prior optimization studies to ensure efficient transfection and reliable modulation of TNIK expression.

### 2.5 RNA extraction and qRT-PCR

Total RNA was extracted 48 h after siRNA transfection using the RNeasy Plus Micro Kit (#74034, QIAGEN), following the manufacturer's instructions. Complementary DNA (cDNA) was synthesized f using the iScript Reverse Transcription Supermix for qRT-PCR (Bio-Rad, #1708841). Each qRT-PCR reaction (10 µl total volume) contained 20 ng of cDNA, 5 µl of iQ SYBR Green Supermix (Bio-Rad), and 0.5 µM of each gene-specific forward and reverse primers. Thermocycling was performed on a QuantStudio RT-PCR System (Applied Biosystems) with the following program: initial denaturation at 95°C for 3 min, followed by 40 cycles of 95°C for 10 s, 60°C for 15 s, and 72°C for 30 s (44, 46, 51, 52). Relative mRNA expression was quantified using the 2^-*ΔΔ*Ct method and normalized to reference gene expression (e.g., *GAPDH*) (53). Primer sequences were synthesized by Sigma-Aldrich and obtained from previously published sources (44, 46, 51, 52).

### 2.6 Mammalian one-hybrid assay to measure ERK5 transcriptional activity

The mammalian one-hybrid assay, a robust and well-established technique employed in our laboratory (3, 11, 44), was used to evaluate ERK5 transcriptional activity. Sub-confluent ECs seeded in 12-well or 6-well plates were transfected with the pG5 luciferase reporter plasmid (pG5-Luc), the pBIND-ERK5 effector construct, and either a CA-MEK5α expression plasmid (pcDNA3.1-CA-MEK5α) (10) or an empty control plasmid (pcDNA3.1). Transfections were performed in Opti-MEM medium (Invitrogen) using Plus-Lipofectamine reagents (1: 1.5 ratio, Plus: Lipofectamine) (3, 11, 44).

In specific experiments, cells were co-transfected with a TNIK expression construct or pre-transfected with siTNIK (50 nM) 48 h prior to plasmid transfection to assess the regulatory effect of TNIK on ERK5 transcriptional activity. After 24 h of post-transfection culture in ECM, cells were lysed using passive lysis buffer (Promega), and luciferase activity was measured using the dual-luciferase reporter assay system (Promega) on a TD-20/20 Luminometer (Turner Designs).

This assay platform has been optimized and validated in multiple experimental contexts to reliably quantify ERK5-dependent transcriptional activation (3, 11, 44).

ERK5 transcriptional activity was quantified as the ratio of firefly luciferase activity (pG5-Luc) to Renilla luciferase activity (pBIND-ERK5), ensuring accurate normalization for transfection efficiency and cell viability. The pG5-Luc reporter plasmid contains five Gal4 DNA-binding sites positioned upstream of a minimal TATA box and the firefly luciferase reporter gene. The pBIND-ERK5 effector construct encodes a fusion of the Gal4 DNA-binding domain fused with full-length ERK5 and includes a Renilla luciferase gene under the control of a constitutive promoter to serve as an internal control. This dual-luciferase system provides high sensitivity and specificity, enabling reliable quantification of ERK5-dependent transcriptional activity across diverse experimental conditions (3, 44).

### 2.7 NFκB activity assay

NF*κ*B activity was quantified using a dual-luciferase reporter assay, a validated and widely adopted method for measuring NF*κ*B-dependent promoter activity (44, 51, 54). The assay employed a firefly luciferase reporter plasmid containing five tandem NF*κ*B-binding sites (pLuc-MCS; Stratagene) and a Renilla luciferase plasmid (pRL-CMV; Promega) used as an internal control for normalization.

ECs were transfected in Opti-MEM Reduced Serum Medium (#31985070; Thermo Fisher Scientific) using DEAE-Dextran (#D9885; Sigma) at a final concentration of 0.375 μg/μl. For each well of a 6-well plate, the transfection mixture was prepared as follows: 3 ml Opti-MEM, 10 μg pLuc-MCS, 0.012 μg pRL-CMV, and 22.5 μl of DEAE-Dextran (50 mg/ml stock). The mixture was incubated for 10 min at 37°C, followed by a PBS wash. The transfection mixture was then applied to the ECs and incubated for 90 min.

Following transfection, cold Opti-MEM supplemented with 5% dimethyl sulfoxide (DMSO) was added for 5 min to enhance transfection efficiency. Cells were then washed with PBS and maintained in ECM for subsequent analyses.

After 24 h, cells were lysed in passive lysis buffer (#E1960; Promega). Firefly and Renilla luciferase activities were measured using the dual-luciferase reporter assay system on a GloMax 20/20 Luminometer (Promega). NF*κ*B activity was expressed as the ratio of firefly to Renilla luciferase luminescence, providing normalization for transfection efficiency. This assay has been routinely optimized and validated in our laboratory, demonstrating high sensitivity, reproducibility, and specificity for quantifying NF*κ*B-dependent activity in ECs (44, 51, 54).

### 2.8 Automated capillary electrophoresis western analysis (Wes)

Protein expression was analyzed using the Protein Simple Wes system, a highly sensitive and reproducible method for quantifying protein levels. Whole-cell lysates were prepared in modified RIPA buffer, and protein concentrations were determined. For each sample, 5 μl of lysate (0.4–1 mg/ml) was loaded onto a 12–230 kDa Separation Module (#SM-W003, Protein Simple) in the Wes system, following the manufacturer's instructions. Rabbit (#DM-001) or Mouse (#DM-002) Detection Modules were utilized for primary antibody detection. Briefly, lysates were mixed with 5× fluorescent master mix containing 200 mM dithiothreitol (DTT), heated at 95°C for 5 min to denature proteins, and loaded onto the separation plate. The plate was sequentially processed with blocking buffer, primary antibodies (diluted in antibody diluent), horseradish peroxidase (HRP)-conjugated secondary antibodies, and luminol-peroxide detection reagents. Antibodies against *β*-actin were multiplexed with target antibodies to serve as loading controls, enabling normalization of protein expression levels across samples.

Capillary electrophoresis was conducted using the system's default settings: separation at 375 V for 25 min, a 5 min blocking step, and 30 min incubations for both primary and secondary antibodies. Protein peaks and identified standards were manually reviewed for accuracy. Data analysis was performed using Compass software (Protein Simple), providing both quantitative and qualitative evaluation of target protein expression. This assay, routinely optimized and validated in our laboratory (44, 50, 51), ensures robust, precise, and reproducible measurements of protein expression levels across experimental conditions.

### 2.9 Flow cytometric analysis of apoptotic cells by Annexin V staining

Flow cytometric analysis of apoptotic cells was performed using Annexin V fluorescein isothiocyanate (FITC) staining, a validated method routinely employed in our laboratory to assess apoptosis. Following siRNA treatment, ECs were washed twice with PBS and harvested using 10 mM EDTA (pH 8.0) at room temperature to preserve cell integrity. Harvested ECs were stained with Annexin V-FITC using the Annexin V-FITC Apoptosis Detection Reagent (#ab14082; Abcam) according to the manufacturer's protocol. Briefly, cell pellets were resuspended in 1× Annexin V Binding Buffer (#ab14084; Abcam) to prepare baseline controls (unstained cells). Annexin V-FITC (#ab14083; Abcam) was added, and samples were incubated for 5 min at room temperature in the dark. Stained cells were immediately analyzed using an Accuri C6 flow cytometer (BD Biosciences), acquiring 10,000 events per sample based on forward and side scatter profiles to ensure robust statistical analysis. Data were processed using FlowJo software (version 10.5.0, FlowJo) (44, 51, 55) to accurately quantify the percentage of apoptotic cells. Inclusion of unstained controls and standardized acquisition settings further enhanced the reliability and reproducibility of the analysis.

### 2.10 Brdu cell proliferation assay

To assess the effect of TNIK-ERK5 signaling on EC proliferation, a bromodeoxyuridine (BrdU) incorporation assay was performed using a commercial BrdU Cell Proliferation Assay Kit (Cell Biolabs, #CBA-251). HUVECs were cultured under standard conditions in endothelial growth medium and seeded into 96-well plates at a density of 0.5 × 10^6^ cells/ml. Upon reaching 70%–80% confluence, cells were transfected with plasmids encoding TNIK WT, DN-MEK5, both TNIK WT and DN-MEK5 (co-transfection), or empty vector (control) using Lipofectamine 2,000 (Thermo Fisher Scientific), following the manufacturer's protocol.

Forty-eight hours after transfection, BrdU labeling solution was added to the culture medium and incubated for 4 h to allow incorporation during DNA synthesis. BrdU incorporation was detected according to the manufacturer's instructions using a primary anti-BrdU antibody followed by an HRP-conjugated secondary antibody. Absorbance was measured at 450 nm using a microplate spectrophotometer, and BrdU incorporation levels were normalized to values from vector-transfected control cells.

## 3 Statistical analysis

Experiments were performed in biological quadruplicates (*n* = 4 independent experiments). Statistical analyses were conducted using GraphPad Prism software (GraphPad Software, LLC). For comparisons between two groups, unpaired two-tailed Student's *t*-tests were used. For comparisons among multiple groups, one-way analysis of variance (ANOVA) followed by appropriate *post hoc* testing was applied. Statistical significance was defined as *p* < 0.05. Quantitative data are presented as mean ± standard error of the mean (SEM).

## 4 Results

### 4.1 TNIK modulates ERK5 transcriptional activity and target gene expression

To investigate whether TNIK regulates ERK5 transcriptional activity, we employed a mammalian one-hybrid assay, an established technique in our laboratory for assessing ERK5 transcriptional regulation (3, 11, 44, 47, 51, 54), alongside qRT-PCR to quantify expression of ERK5 target genes (48). Human ECs were transfected with TNIK-specific siRNA (siTNIK) or control siRNA (siCont). Representative microscopic images from our previous publication (46) demonstrate no significant morphological differences between ECs transfected with siCont or siTNIK up to 120 h post-transfection. These findings confirm that siRNA transfection did not induce gross morphological changes or cytotoxic effects, validating the integrity of the experimental system.

**Figure 1:**
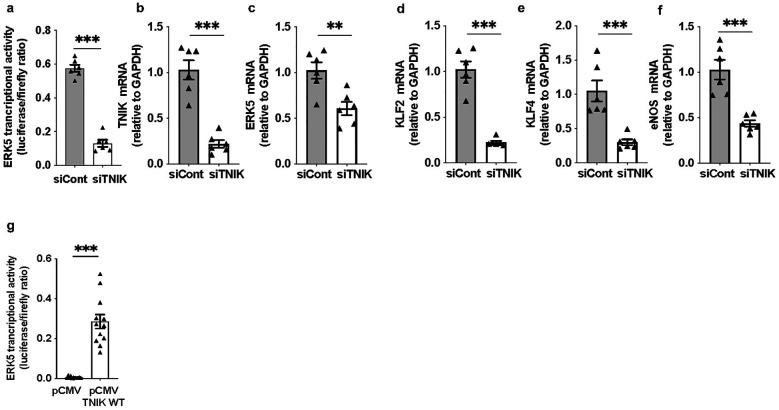
TNIK regulates ERK5 transcriptional activity and target gene expression in ECs: **(a)** TNIK knockdown suppresses ERK5 transcriptional activity: HAECs were seeded at 50% confluence in 12-well-plates and transfected with siTNIK or siCont (50 µM each). After 48 h, cells were co-transfected with the pG5-Luc luciferase reporter (0.3 µg/well) and pBIND-ERK5 effector plasmid (0.3 µg/well). Luciferase activity was measured 24 h post-co-transfection using a dual-luciferase assay. ERK5 transcriptional activity was reduced in TNIK-depleted cells compared to controls. **(b–f)** TNIK knockdown decreases ERK5 target gene expression: HAECs were seeded at 50% confluence in 6-well-plates and transfected with siTNIK or siCont (50 µM each). Total RNA was isolated 48 h post-transfection, and transcript levels of *TNIK*, *ERK5*, and ERK5 target genes (*KLF2, KLF4*, and *eNOS*) were quantified by qRT-PCR, normalized to *GAPDH*. TNIK knockdown resulted in a reduction in expression of all assessed transcripts. **(g)** TNIK overexpression enhances ERK5 transcriptional activity: HUVECs were seeded at 50% confluence on 12-well-plates and co-transfected with pG5-Luc (0.3 µg/well), pBIND-ERK5 (0.3 µg/well), and either an empty control vector (pCMV, 0.3 µg/well) or a TNIK WT expression construct (TNIK WT, 0.3 µg/well). Luciferase activity assessed 24 h post-transfection, demonstrated an increase in ERK5 transcriptional activity in TNIK-overexpressing cells compared to vector controls. Data represent results from three independent experiments. Statistical comparisons were performed using unpaired two-tailed Student's *t*-tests. Significance thresholds: ***p < 0.001, **p < 0.01. Data presented as mean ± SEM. Sample sizes: *n* = 6 **(a–f)**, *n* = 12 **(g)**.

Forty-eight hours after siRNA transfection, cells were co-transfected with the pG5-Luc reporter and pBIND-ERK5 effector plasmids (44). Luciferase activity measured 24 h later revealed a reduction in ERK5 transcriptional activity in TNIK-depleted cells [Figure 1a]. This was accompanied by a marked decrease in mRNA levels of canonical ERK5 target genes, including *KLF2, KLF4,* and *eNOS* [Figures 1b–f], confirming the essential role of TNIK in maintaining ERK5-dependent transcriptional programs in ECs.

To substantiate these observations, gain-of-function experiments were performed. ECs were co-transfected with pG5-Luc and pBIND-ERK5, along with either a control plasmid (pCMV-2B) or a wild-type TNIK expression construct (TNIK WT). Overexpression of TNIK WT increased ERK5 transcriptional activity compared to vector control (Figure 1g), corroborating the function of TNIK as a positive regulator of ERK5-mediated transcriptional activation.

### 4.2 TNIK knockdown does not affect CA-MEK5α-driven ERK5 transcriptional activation

**Figure 2:**
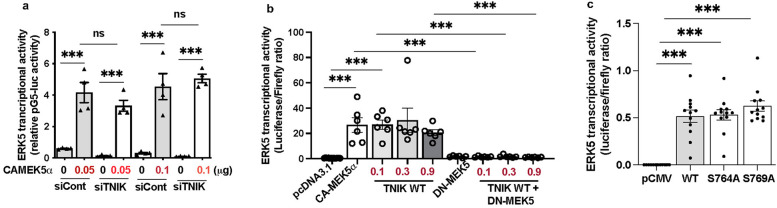
TNIK regulates ERK5 transcriptional activity through both MEK5-dependent and independent mechanisms: **(a)** TNIK knockdown does not attenuate ERK5 transcriptional activation by CA-MEK5α: HUVECs were seeded at 50% confluence in 12-well-plates and transfected with either siTNIK or siCont (50 µM each). After 48 h, cells were co-transfected with pG5-Luc (0.3 µg/well), pBIND-ERK5 (0.3 µg/well), and increasing doses of CA-MEK5α (0.05 or 0.1 µg/well). Luciferase activity measured 24 h post- transfection revealed that CA-MEK5α increases ERK5 transcriptional activity at both doses, unaffected by TNIK knockdown, indicating that TNIK is dispensable under conditions of sustained MEK5 activation. **(b)** TNIK-induced ERK5 transcriptional activation requires functional MEK5: HUVECs seeded on 12-well plates at 50% confluence were co-transfected with pG5-Luc (0.3 µg/well), pBIND-ERK5 (0.3 µg/well), and the indicated constructs: pcDNA3.1 (empty vector, 0.3 µg/well), CA-MEK5α (0.3 µg/well), TNIK WT (0.1 µg, 0.3 µg, or 0.9 µg/well), DN-MEK5 (0.3 µg/well), or TNIK WT plus DN-MEK5. TNIK WT enhanced ERK5 transcriptional activity comparably to CA-MEK5*α*; however, co-expression with DN-MEK5 abrogated this effect, indicating that TNIK requires intact MEK5 signaling to potentiate ERK5 transcriptional activation. (**c**) TNIK S764A and S769A mutants activate ERK5 transcriptional activity similarly to WT: HUVECs seeded at 50% confluence in 12-well plates were transfected with pG5-Luc (0.3 µg/well), pBIND-ERK5 (0.3 µg/well), and either empty vector (pCMV-2B), TNIK WT, or mutants TNIK S764A and TNIK S769A. Luciferase assays measured 24 h post-transfection revealed that both mutants increased ERK5 transcriptional activity similarly to TNIK WT, suggesting that TNIK regulates ERK5 transcriptional activity independently of S764 and S769 phosphorylation. Data represent three independent experiments. Statistical comparisons were performed using one-way ANOVA followed by Bonferroni *post hoc* testing. Significance thresholds: ***p < 0.001 vs. control. Results are expressed as mean ± SEM. Sample sizes: *n* = 4 **(a)**, *n* = 6 **(b)**, *n* = 12 **(c)**.

To assess whether TNIK modulates ERK5 transcriptional activity through MEK5 signaling, we evaluated ERK5 transcriptional activation in the presence of constitutively active MEK5 (CA-MEK5α) (10), with or without TNIK knockdown. As expected, CA-MEK5α enhanced ERK5 transcriptional activity at both 0.05 and 0.1 µg plasmid concentrations (Figure 2a). Notably, TNIK knockdown did not attenuate CA-MEK5α-driven ERK5 transcriptional activation (Figure 2a), indicating that TNIK is dispensable under conditions of sustained MEK5 activity. These findings suggest that MEK5 can maintain ERK5 transcriptional activity independently of TNIK when constitutively active.

To further elucidate the relationship between TNIK and MEK5 in regulating ERK5 signaling, we systematically tested the following conditions: control plasmid (pcDNA3.1), CA-MEK5*α*, TNIK WT, dominant-negative MEK5 (DN-MEK5, a construct that inhibits MEK5 activity), and co-expression of TNIK WT with DN-MEK5. As anticipated, CA-MEK5*α* increased ERK5 transcriptional activity, whereas DN-MEK5 alone failed to stimulate activation. Overexpression of TNIK WT similarly promoted ERK5 transcriptional activity to a level comparable to that archived with CA-MEK5*α* (Figure 2b). However, co-expression of DN-MEK5 abolished TNIK WT-mediated ERK5 transcriptional activation (Figure 2b), indicating that functional MEK5 is indispensable for TNIK-dependent regulation of ERK5 transcriptional signaling.

Collectively, these findings indicate that TNIK operates within a MEK5-dependent regulatory framework. Under physiological conditions, TNIK likely functions upstream of, or in concert with, MEK5 to facilitate ERK5 transcriptional activation. However, when MEK5 is constitutively active, its dominant signaling capacity can bypass the requirement for TNIK.

### 4.3 TNIK regulation of ERK5 through non-canonical mechanisms

Previous findings demonstrated that ponatinib suppresses ERK5 transcriptional activity and downregulates its target genes (*KLF2, KLF4, eNOS*) even under conditions of constitutive MEK5 activation (CA-MEK5α) (44), and that ponatinib also inhibits TNIK (45). Notably, ponatinib suppresses ERK5 transcriptional activity without affecting ERK5 phosphorylation at the T218/Y220 residues (44), suggesting that TNIK may regulate ERK5 through non-canonical mechanisms. One hypothesis is that TNIK either competes with MEK5 for ERK5 binding or facilitates ERK5 activation though a structural or scaffolding role, independent of its kinase activity.

Supporting this hypothesis, pitavastatin has been shown to directly activate ERK5 kinase activity in a cell-free system and increase *KLF2* expression in an MEK5-independent manner (47). Importantly, TNIK knockdown abolished pitavastatin-induced ERK5 transcriptional activation (Supplementary Figure S1), indicating that TNIK contributes to ERK5 function through mechanisms beyond canonical MEK5-mediated phosphorylation.

### 4.4 TNIK regulation of ERK5 transcriptional activity independent of S764 and S769 phosphorylation

To determine whether phosphorylation at TNIK residues S764 and S769 is necessary for its regulatory function, site-directed mutants (S764A and S769A) were generated. Overexpression of TNIK WT, S764A, and S769A each enhanced ERK5 transcriptional activity to comparable levels (Figure 2c). These findings indicate that phosphorylation at S764 and S769 is not required for TNIK-mediated regulation of ERK5 transcriptional activity, suggesting that TNIK exerts its effects through a mechanism independent of phosphorylation at these sites, and possibly independent of its intrinsic kinase activity.

### 4.5 TNIK knockdown suppresses ERK5 T218/Y220 phosphorylation induced by l-flow

**Figure 3:**
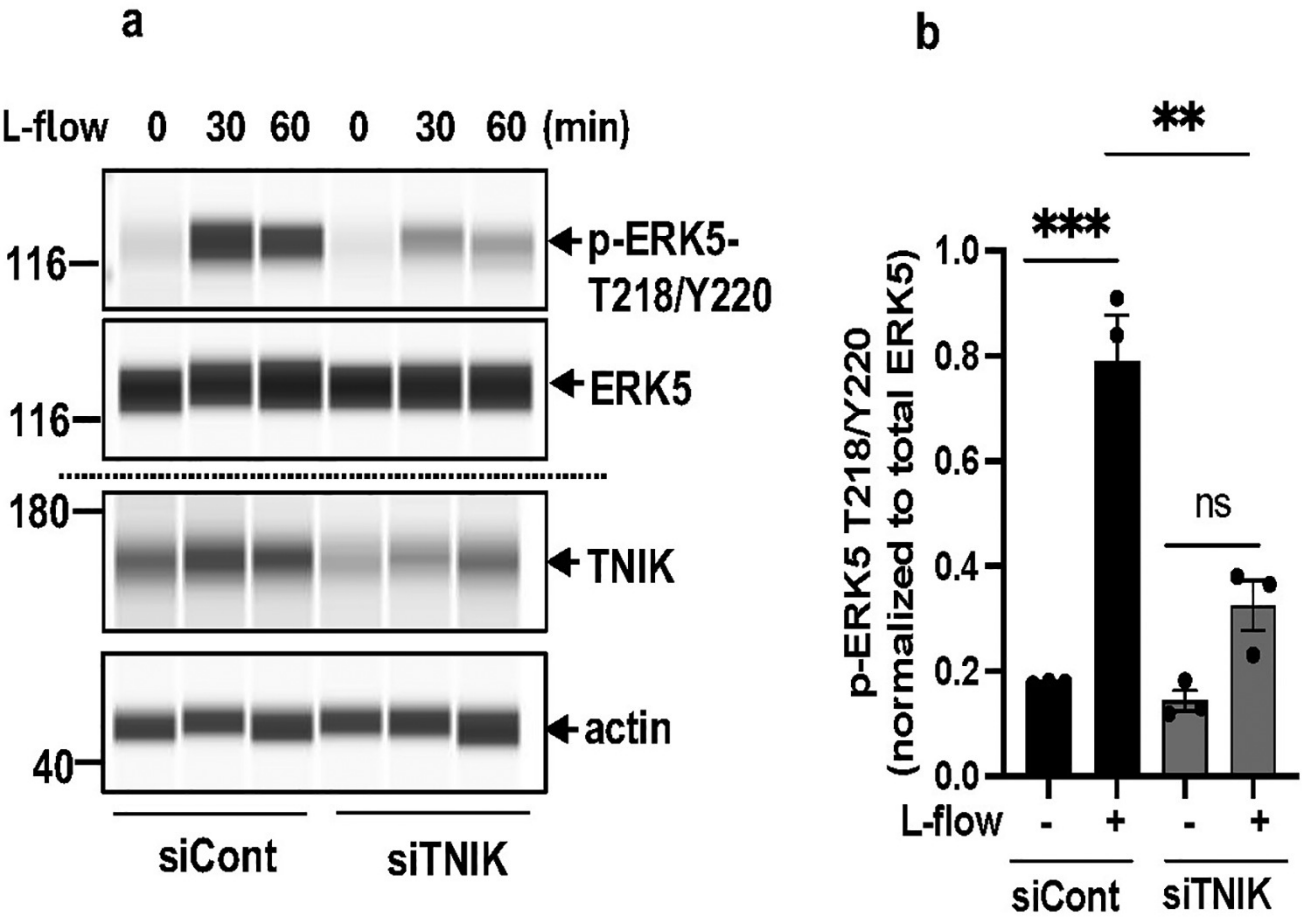
TNIK knockdown inhibits l-flow-mediated ERK5 T218/Y220 phosphorylation: **(a)** HAECs were seeded at 50% confluence in 10 cm plates and transfected with siTNIK or siCont (50 µM each). After 48 h, cells were exposed to l-flow, and ERK5 T218/Y220 phosphorylation was assessed by automated capillary electrophoresis (WES; Protein Simple). TNIK knockdown reduced l-flow-induced ERK5 T218/Y220 phosphorylation compared to control. **(b)** Quantification of phosphorylated ERK5 T218/Y220 relative to total ERK5 was performed 30 min after l-flow stimulation. TNIK-depleted cells exhibited a reduction in ERK5 T218/Y220 phosphorylation. Data represent results from three independent experiments. Statistical comparisons were performed using an unpaired two-tailed Student's *t*-test. Significance thresholds: **p < 0.01, ***p < 0.001 vs. control. Results are expressed as mean ± SEM.

Canonical MEK5-dependent ERK5 activation involves phosphorylation at T218/Y220 by MEK5, leading to activation of the downstream transcriptional factor MEF2 and regulation of *KLF2, KLF4*, and *eNOS (*1, 2, 4–8, 12, 14–16, 56). To evaluate the impact of TNIK knockdown on ERK5 T218/Y220 phosphorylation, ECs transfected with siTNIK or siCont were exposed to l-flow, a physiological stimulus known to activate ERK5 signaling. Protein analysis using the Wes capillary-based immunoassay system revealed a reduction in l-flow-induced ERK5 T218/Y220 phosphorylation (44) in TNIK-depleted ECs compared to controls (Figures 3a,b). These findings suggest that TNIK functions upstream of, or in concert with, MEK5 in modulating ERK5 T218/Y220 phosphorylation and activation in response to physiological hemodynamic forces.

### 4.6 TNIK knockdown increases NFκB activity and apoptosis in ECs

**Figure 4:**
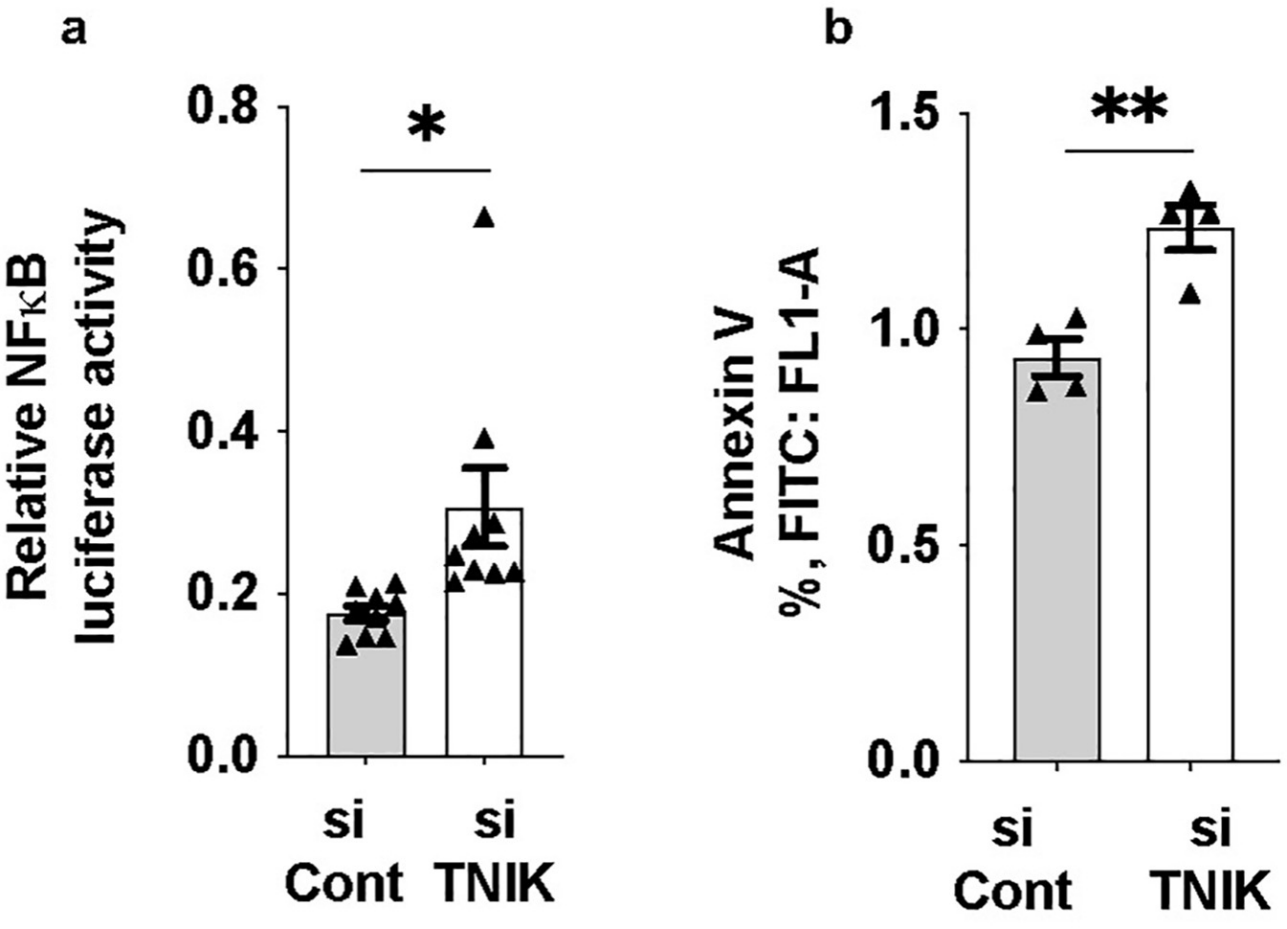
TNIK knockdown increases NF-κB activity and apoptosis in ECs: **(a)** HAECs were seeded at 50% confluence in 6-well-plates and transfected with siTNIK or siCont (50 µM each). After 48 h, cells were co-transfected with an NF-κB luciferase reporter plasmid and pRL-CMV vector for normalization (44). Luciferase activity was measured 24 h post-transfection and revealed an increase in NF-*κ*B activity in TNIK-deleted cells compared to controls (*p < 0.05). **(b)** Apoptosis was assessed 48 h after siRNA transfection using Annexin V staining and flow cytometry (54). TNIK knockdown resulted in an increase in the percentage of Annexin V-positive cells compared to control (**p < 0.01). Data represent results from three independent experiments. Statistical comparisons were performed using unpaired two-tailed Student's t-tests. Significance thresholds: **p < 0.01; *p < 0.05. Data are presented as mean ± SEM. Sample sizes: n = 9 **(a)**, *n* = 4 **(b)**.

Reduced ERK5 transcriptional activity has been closely associated with increased inflammation, a hallmark of EC dysfunction (3). To investigate the functional consequence of TNIK depletion, we assessed NF*κ*B activity and apoptosis. TNIK knockdown led to an increase in NF*κ*B activity (Figure 4a) and a concomitant rise in apoptosis levels (Figure 4b), underscoring TNIK's critical role in maintaining EC viability and homeostatic signaling. These findings are consistent with the role of TNIK in sustaining ERK5 transcriptional activity (Figure 1a) and highlight its involvement in modulating both ERK5 and NF*κ*B signaling pathways, which are essential for vascular homeostasis.

Interestingly, despite enhanced NF*κ*B activity (Figure 4a), TNIK knockdown did not upregulate the mRNA expression of canonical NF*κ*B target genes, including adhesion molecules (*VCAM1, ICAM1*; Supplementary Figures S2a,b), cytokines (*TNFα, IL6*; Supplementary Figures S2c,d), and the chemokine *MCP1* (Supplementary Figure S2e). This paradox suggests that TNIK depletion activates NF*κ*B signaling at the level of nuclear translocation or upstream pathways without concomitant transcriptional induction of downstream target genes, revealing a complex regulatory relationship between TNIK, NF*κ*B, and transcriptional output in ECs.

### 4.7 TNIK regulates EC proliferation through a MEK5-dependent mechanism

**Figure 5:**
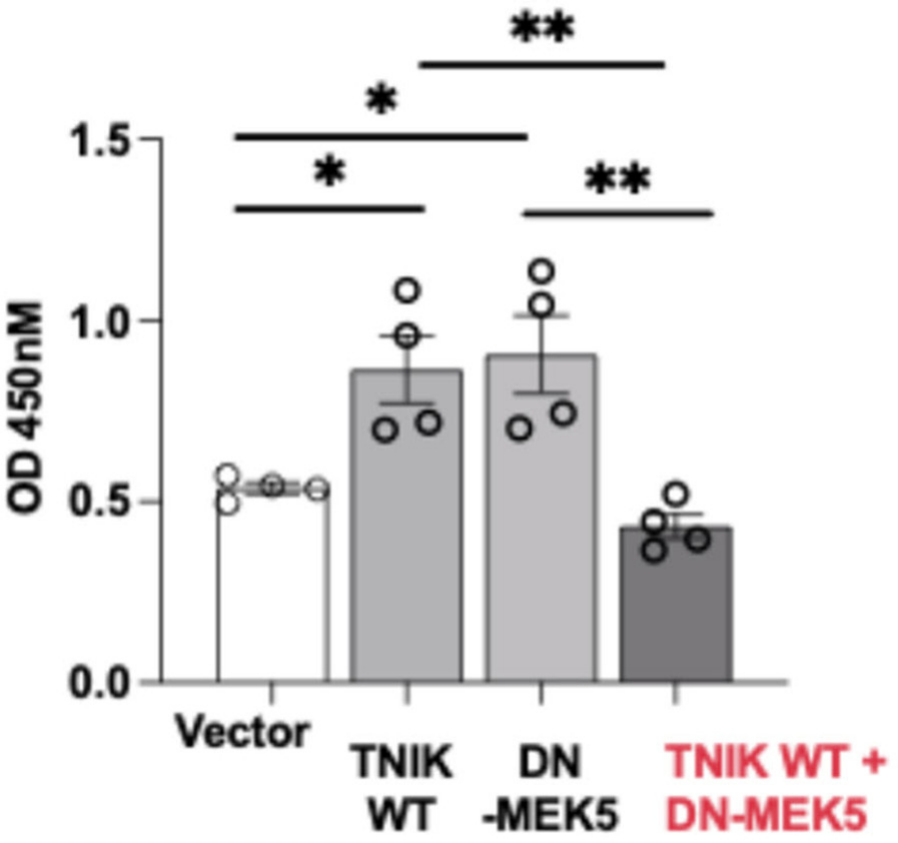
TNIK promotes EC proliferation through a MEK5-dependent mechanism: BrdU incorporation assays were performed in HUVECs to evaluate the effects of TNIK WT and DN-MEK5 on cell proliferation. Overexpression of TNIK WT or DN-MEK5 individually increased BrdU incorporation relative to vector control. Co-transfection of TNIK WT and DN-MEK5 attenuated this proliferative effect, indicating that intact MEK5 signaling is required for TNIK-mediated promotion of EC proliferation. Data are presented as mean ± SEM from four independent experiments. Statistical comparisons were performed using one-way ANOVA followed by *post hoc* testing; Significance thresholds **p < 0.01; *p < 0.05.

Given the observed increase in apoptosis following TNIK depletion, we next examined whether TNIK signaling also influences EC proliferation. BrdU incorporation assays revealed that overexpression of TNIK WT enhanced proliferation compared to vector control (Figure 5). Unexpectedly, DN-MEK5 overexpression also increased BrdU incorporation relative to control. This observation stands in contrast to prior reports wherein MEK5-ERK5 pathway inhibition is generally associated with attenuated endothelial proliferation. To our knowledge, no published study has described a proliferation-enhancing effect of DN-MEK5 expression. The mechanism basis for this paradox remains unresolved; it may reflect an off-target response, altered cell-cycle regulation, or a context-specific compensatory adaptation. Notably, co-expression of TNIK WT with DN-MEK5 abolished the proliferative effect observed with TNIK WT alone, indicating that functional MEK5 signaling is required for TNIK-mediated promotion of EC proliferation. Collectively, these findings support a model in which TNIK contributes to endothelial survival and proliferative capacity primarily through a MEK5-dependent mechanism, reinforcing its role in the maintenance of vascular homeostasis.

## 5 Discussion

ERK5 plays multifaceted roles in cancer pathogenesis, with its activation mechanisms differing across tumor types (57). In neuroblastoma and mesothelioma, ERK5 is activated by PI3K/AKT signaling; in breast cancer, STAT3 enhances MEK5 expression; and in renal epithelial cells, TGFβ1-induced MEK5/ERK5 activation occurs through ALK and p38 MAPK, facilitating MEF2C-mediated transcription. These upstream signals promote tumor cell survival, differentiation, and proliferation. These findings support the investigation of ERK5 as a potential therapeutic target in oncological and non-oncological contexts, although its full biological spectrum remains incompletely defined (7, 58).

Beyond cancer, ERK5 is essential for vascular homeostasis. EC-specific deletion of ERK5 in mice impairs vascular reactivity, increases leukocyte rolling, and accelerates atherosclerosis (3). In cultured ECs, l-flow activates ERK5, inducing anti-inflammatory phenotypes through upregulation of protective genes (e.g., *KLF2, KLF4*, *eNOS)* amd suppression of pro-inflammatory mediators (*VCAM1, ICAM1, E-selectin)* (3).

Previous studies, including our own (44, 45), showed that the multi-kinase inhibitor ponatinib suppresses ERK5 transcriptional activity (44) and inhibits TNIK (45), suggesting a possible upstream regulatory role for TNIK. To minimize off-target effects of small-molecule inhibitors (45), we employed an siRNA-mediated TNIK knockdown approach. This builds upon our prior findings that TNIK modulates interferon and cytokine signaling in ECs (46).

Our results demonstrate that TNIK knockdown reduces ERK5 transcriptional activity and decreases expression of canonical ERK5 targets (*KLF2, KLF4, eNOS)* (Figure 1). Overexpression of CA-MEK5*α* rescued ERK5 transcriptional activity in TNIK-depleted cells (Figure 2a), suggesting that TNIK functions upstream, or in cooperative with, MEK5 to regulate ERK5 transcriptional activity. Furthermore, TNIK overexpression alone increased ERK5 transcriptional activity to levels comparable to CA-MEK5*α*, whereas co-expression with DN-MEK5 abolished this effect (Figure 2b). These findings establish that TNIK regulates ERK5 through a MEK5-dependent mechanism.

We further examined whether this mechanism requires TNIK phosphorylation at key residues. Mutant TNIK constructs lacking phosphorylation at S764 or S769 (S764A and S769A) retained the ability to induce ERK5 transcriptional activity (Figure 2c), indicating that TNIK modulates ERK5 transcriptional activity independently of phosphorylation at these sites and potentially independently of its kinase function. This aligns with studies showing that TNIK can regulate signaling pathways through ubiquitination rather than phosphorylation (59).

To determine whether TNIK is required for canonical MEK5-mediated ERK5 activation at the phosphorylation level, we assessed ERK5 phosphorylation at T218/Y220 following l-flow stimulation. TNIK knockdown suppressed l-flow-induced ERK5 T218/Y220 phosphorylation (Figure 3), demonstrating that TNIK is necessary for physiological activation of the MEK5-ERK5 pathway under flow conditions.

Beyond its influence on ERK5 signaling, TNIK depletion affected inflammatory and survival signaling in ECs. TNIK knockdown increased NF*κ*B activity (Figure 4a) but this was not accompanied by increased expression of canonical NF*κ*B target genes (*VCAM1*, *ICAM1*, *TNFα*, *IL6*, *MCP1)* (Supplementary Figures S2a–e). This mechanistic paradox may reflect a requirement for TNIK not only in NF*κ*B activation but also in transcriptional competence- possibly through modulation of chromatin accessibility or co-activator recruitment. Alternatively, a shift between p65-driven canonical and p52/RelB-driven non-canonical NF*κ*B pathways (59–70) could result in incomplete or aberrant transcriptional responses. Our findings are consistent with known roles of TNIK in facilitating TRAF6 ubiquitination and promoting IKK complex assembly via interaction with TAB2, a process essential for canonical NF-*κ*B signaling (59, 68–70). Together, the data support a model in which TNIK integrates ERK5 and NF-*κ*B signaling to regulate endothelial inflammatory tone.

We also found that TNIK supports EC proliferation through a MEK5-dependent mechanism. Overexpression of TNIK increased BrdU incorporation in ECs, an effect abolished by co-expression of DN-MEK5 (Figure 5). Notably, DN-MEK5 alone also increased BrdU incorporation, possibly due to a compensatory activation of alternative mitogenic pathways (e.g., PI3K-AKT) in contexts of ERK5 inhibition.

Ponatinib suppresses ERK5 transcriptional activity without inhibiting ERK5 T218/Y220 phosphorylation (44). In TNIK-depleted ECs, pitavastatin failed to stimulate ERK5 transcriptional activity (Supplementary Figure S1), suggesting that TNIK may act as a scaffold or structural facilitator of ERK5 function, possibly by stabilizing the ERK5 transcriptional complex or enhancing nuclear translocation.

Building on our earlier observation that TNIK knockdown impairs interferon responses (46), the present data further support a broader role for TNIK as a multifunctional scaffold. This concept is aligned with its established involvement in Wnt/β-catenin signaling (18–21, 27, 41), interferon modulation, and cytokine signaling (46). Notably, our findings now implicate TNIK in ER stress pathways: its knockdown reduced expression of *CHOP,* a transcriptional effector of ER stress-induced apoptosis (71, 72), and altered splicing of *XBP1*-decreasing total *XBP1* while trending toward increased spliced *XBP1* (Supplementary Figures S2f–h). These changes may reflect a compensatory adaptation to ER stress, helping reconcile the paradox of suppressed NF-κB target gene expression yet elevated NF-κB activity. Since CHOP also modulates NF*κ*B signaling (73, 74) and mediate ER stress-induced apoptosis (71, 72, 75, 76), its downregulation may serve to limit apoptosis and preserve EC viability under stress.

A plausible mechanistic model may involve TNIK interaction with MAGI1, a PDZ domain–containing scaffold protein previously shown to regulate ER stress signaling and atherosclerosis in ECs (51, 77). MAGI1 is regulated by p90RSK (51)-a kinase that inhibits ERK5 activity (3), suggesting a signaling module composed of TNIK, MAGI1, p90RSK, and ERK5. Such a complex may integrate inflammatory signals in ECs.

Importantly, TNIK's role extends beyond the vascular system. It is implicated in neurodevelopment and psychiatric disorders (20, 29–41). TNIK is found in postsynaptic density complexes enriched for risk genes associated with autism spectrum disorder, intellectual disability, developmental delay, and schizophrenia (78). These interactions likely involve PDZ-binding scaffold proteins such as MAGI1 (51, 77, 78). Our prior work identified MAGI1 as a key regulator of ER stress in ECs (51), supporting the idea that TNIK-MAGI1 interactions may mediate broader regulatory functions in both vascular and neural contexts.

Collectively, our findings expand TNIK's known repertoire beyond cytoskeletal dynamics and neurodevelopment, revealing it as an integrative hub coordinating ERK5 transcriptional activity, NF*κ*B signaling, ER stress responses, and EC proliferation and survival. These data support a model in which TNIK serves as a multifunctional scaffold critical for endothelial homeostasis and vascular integrity.

While our *in vitro* data offer important mechanistic insights, they may not fully recapitulate *in vivo* vascular complexity. The molecular interfaces among TNIK, ERK5, and MAGI1 remain to be precisely defined. Future studies using EC-specific TNIK knockout models, in combination with biochemical methods such as proximity ligation, co-immunoprecipitation, and super-resolution imaging, will be crucial for mapping TNIK's interaction networks. Furthermore, investigating the role of RAP2a, a Ras GTPase implicated as a TNIK effector, may offer further insight. It will be important to determine whether RAP2a-driven farnesylation or membrane localization modulates TNIK-ERK5 interactions.

In conclusion, our study identifies TNIK as a central regulator of ERK5 activity, NF*κ*B signaling, ER stress adaptation, and EC proliferation and survival. These results position TNIK not only as a kinase in Wnt/*β*-catenin signaling but as a multifunctional scaffold essential for vascular homeostasis. TNIK thus emerges as a promising therapeutic target for vascular diseases involving EC dysfunction, chronic inflammation, and potentially broader immunologic and neuropsychiatric pathologies.

## Data Availability

The original contributions presented in the study are included in the article/Supplementary Material, further inquiries can be directed to the corresponding authors.

## Supplementary material

Supplementary Figure 1 TNIK knockdown inhibits Pitavastatin (Pita)-driven ERK5 transcriptional activation: HAECs were seeded at 50% confluence in 6-well-plates and transfected with siTNIK or siCont (50 µM each). After 48 hours, cells were co-transfected with pG5-Luc (0.6 µg/well) and pBIND-ERK5 (0.6 µg/well). Twenty-four hours later, cells were treated with Pitavastatin (1 µM), for an additional 24 hours. Luciferase activity was then measured to assess ERK5 transcriptional activity. Pitavastatin increased ERK5 transcriptional activity in control cells, an effect that was abolished by TNIK knockdown. Data represent results from three independent experiments. Statistical comparisons were performed using one-way ANOVA followed by Bonferroni post hoc testing. Significance thresholds: ***p* < 0.01, **p* < 0.05, ns (not significant). Data are presented as mean ± SEM. Sample sizes: *n* = 4.

Supplementary Figure 2 TNIK knockdown decreases mRNA levels of key NF-κB target genes. HAECs were seeded at 50% confluence in 6-well-plates and transfected with siTNIK or siCont (50 µM each). After 48 hours, cells were harvested, and total RNA was extracted. Transcript levels of canonical NF-κB target genes (*VCAM1,ICAM1, TNF, IL-6, MCP1),* ER stress marker *CHOP,* and XBP1 isoforms (total *XBP1* and *spliced XBP1*) were quantified by qRT-PCR, normalized to *GAPDH*. TNIK knockdown reduced expression of these genes compared to control. Data represent results from three independent experiments. Statistical comparisons were performed using unpaired two-tailed Student's t-tests. Significance thresholds: ****p* < 0.001, ***p* < 0.01. Data are presented as mean ± SEM. Sample sizes: *n* = 6.
