# Supplementary figures and images for: TNIK-driven regulation of ERK5 transcriptional activity in endothelial cells
Source: Front Cardiovasc Med. 2025 Jul 2;12:1526676. doi: 10.3389/fcvm.2025.1526676 (PMC12263683; doi:10.3389/fcvm.2025.1526676)

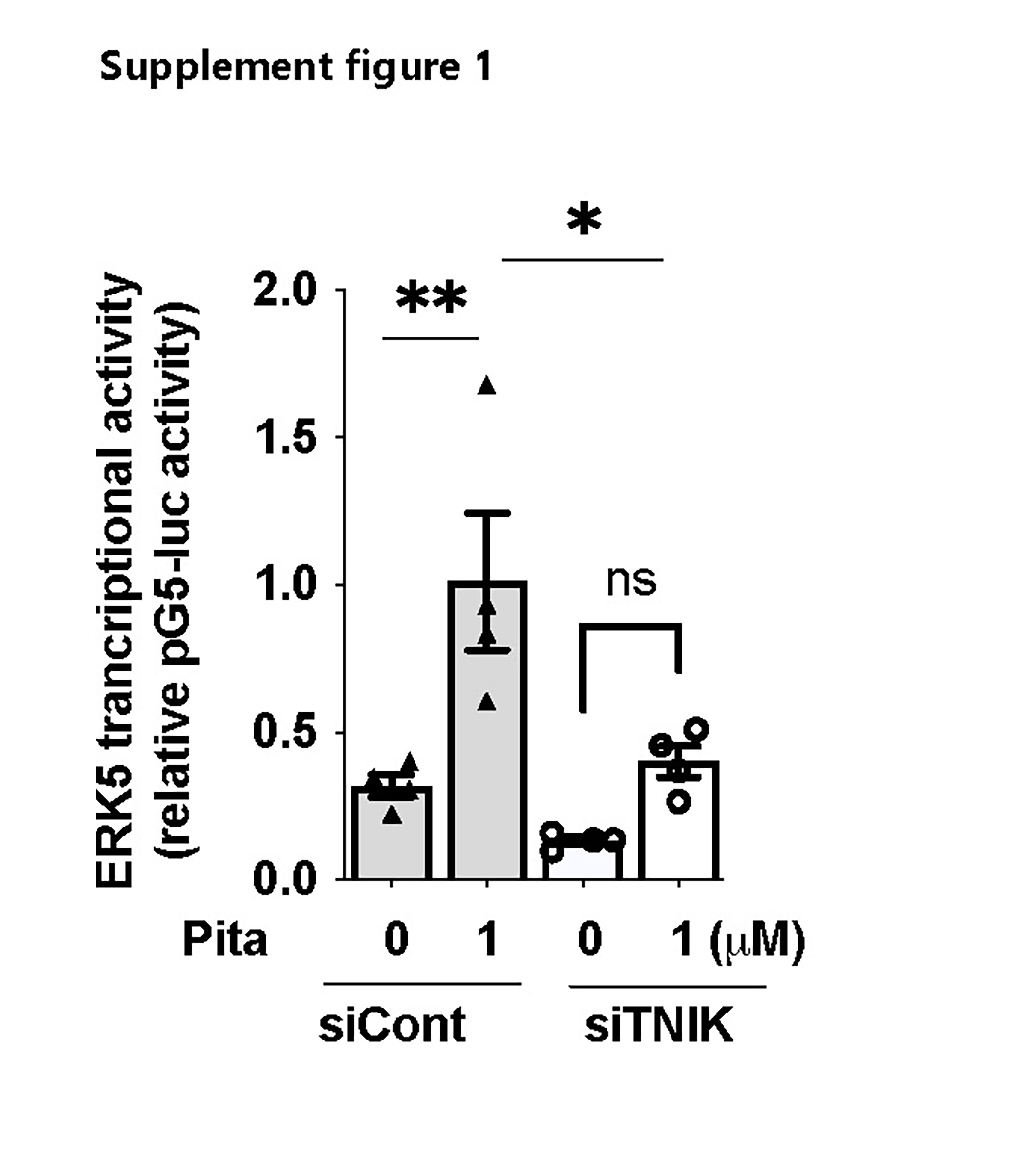

Supplement: Supplementary Figure 1 — TNIK knockdown inhibits Pitavastatin (Pita)-driven ERK5 transcriptional activation: HAECs were seeded at 50% confluence in 6-well-plates and transfected with siTNIK or siCont (50 µM each). After 48 hours, cells were co-transfected with pG5-Luc (0.6 µg/well) and pBIND-ERK5 (0.6 µg/well). Twenty-four hours later, cells were treated with Pitavastatin (1 µM), for an additional 24 hours. Luciferase activity was then measured to assess ERK5 transcriptional activity. Pitavastatin increased ERK5 transcriptional activity in control cells, an effect that was abolished by TNIK knockdown. Data represent results from three independent experiments. Statistical comparisons were performed using one-way ANOVA followed by Bonferroni post hoc testing. Significance thresholds: **p < 0.01, *p < 0.05, ns (not significant). Data are presented as mean ± SEM. Sample sizes: n = 4. [file Image1.jpeg]

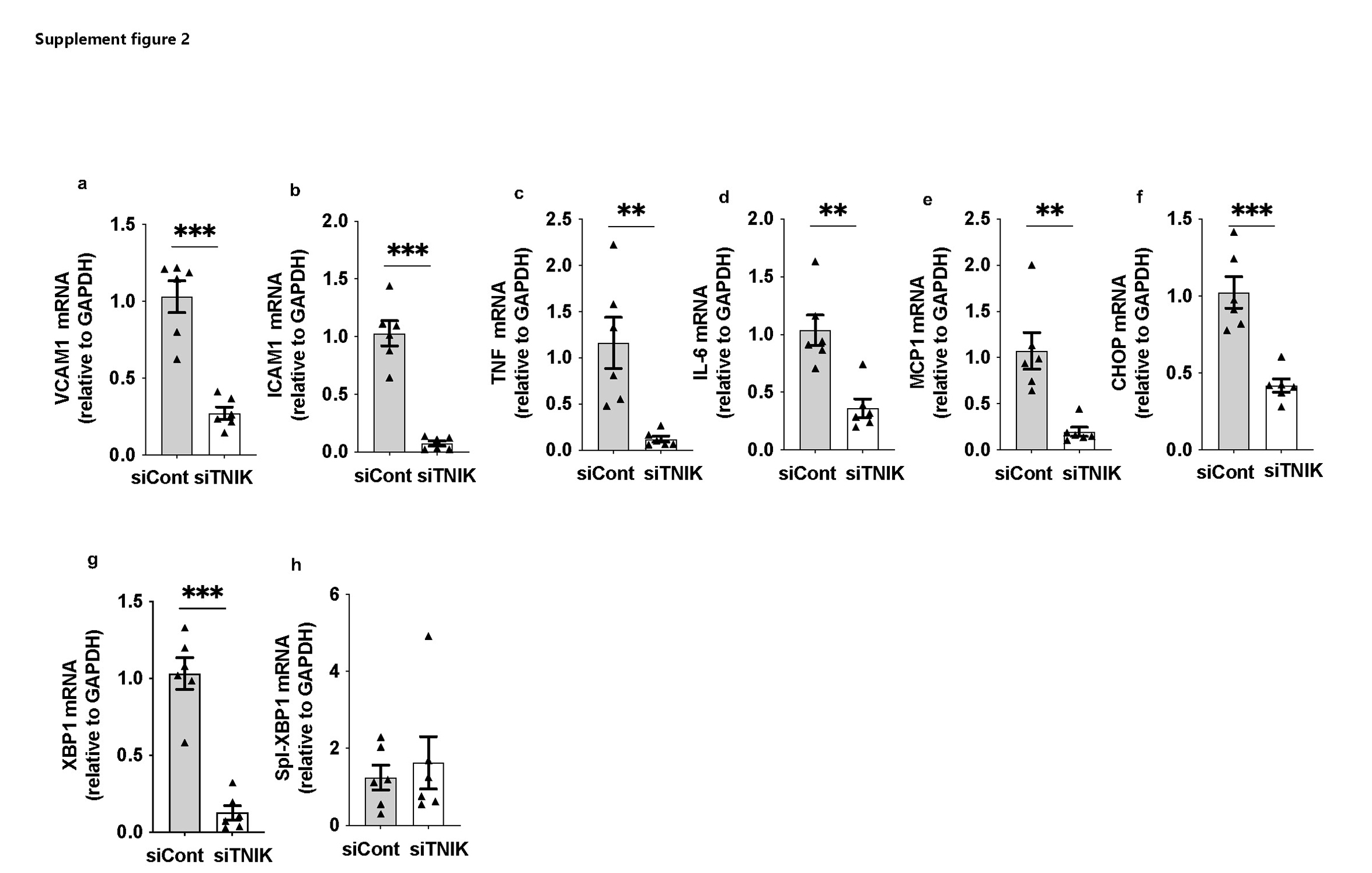

Supplement: Supplementary Figure 2 — TNIK knockdown decreases mRNA levels of key NF-κB target genes. HAECs were seeded at 50% confluence in 6-well-plates and transfected with siTNIK or siCont (50 µM each). After 48 hours, cells were harvested, and total RNA was extracted. Transcript levels of canonical NF-κB target genes (VCAM1,ICAM1, TNF, IL-6, MCP1), ER stress marker CHOP, and XBP1 isoforms (total XBP1 and spliced XBP1) were quantified by qRT-PCR, normalized to GAPDH. TNIK knockdown reduced expression of these genes compared to control. Data represent results from three independent experiments. Statistical comparisons were performed using unpaired two-tailed Student's t-tests. Significance thresholds: ***p < 0.001, **p < 0.01. Data are presented as mean ± SEM. Sample sizes: n = 6. [file Image2.jpeg]
